# Applying human-centered design to the construction of a cirrhosis management clinical decision support system

**DOI:** 10.1097/HC9.0000000000000394

**Published:** 2024-02-26

**Authors:** Jin Ge, Ana Buenaventura, Beth Berrean, Jory Purvis, Valy Fontil, Jennifer C. Lai, Mark J. Pletcher

**Affiliations:** 1 Department of Medicine, Division of Gastroenterology and Hepatology, University of California—San Francisco, San Francisco, California, USA; 2 School of Medicine Technology Services, University of California—San Francisco, San Francisco, California, USA; 3 Family Health Centers, NYU-Langone Medical Center, Brooklyn, New York, USA; 4 Department of Epidemiology and Biostatistics, University of California—San Francisco, San Francisco, California, USA

## Abstract

**Background:**

Electronic health record (EHR)-based clinical decision support is a scalable way to help standardize clinical care. Clinical decision support systems have not been extensively investigated in cirrhosis management. Human-centered design (HCD) is an approach that engages with potential users in intervention development. In this study, we applied HCD to design the features and interface for a clinical decision support system for cirrhosis management, called *CirrhosisRx*.

**Methods:**

We conducted technical feasibility assessments to construct a visual blueprint that outlines the basic features of the interface. We then convened collaborative-design workshops with generalist and specialist clinicians. We elicited current workflows for cirrhosis management, assessed gaps in existing EHR systems, evaluated potential features, and refined the design prototype for *CirrhosisRx*. At the conclusion of each workshop, we analyzed recordings and transcripts.

**Results:**

Workshop feedback showed that the aggregation of relevant clinical data into 6 cirrhosis decompensation domains (defined as common inpatient clinical scenarios) was the most important feature. Automatic inference of clinical events from EHR data, such as gastrointestinal bleeding from hemoglobin changes, was not accepted due to accuracy concerns. Visualizations for risk stratification scores were deemed not necessary. Lastly, the HCD co-design workshops allowed us to identify the target user population (generalists).

**Conclusions:**

This is one of the first applications of HCD to design the features and interface for an electronic intervention for cirrhosis management. The HCD process altered features, modified the design interface, and likely improved *CirrhosisRx*’s overall usability. The finalized design for *CirrhosisRx* proceeded to development and production and will be tested for effectiveness in a pragmatic randomized controlled trial. This work provides a model for the creation of other EHR-based interventions in hepatology care.

## Graphical abstract

**Figure:**
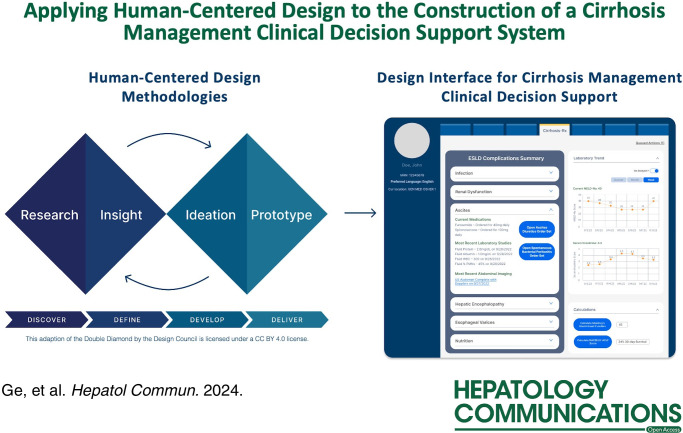

## INTRODUCTION

Cirrhosis and its complications are associated with significant morbidity, mortality, and health care utilization.^1^,^2^ Moreover, prior studies have indicated significant differences in inpatient mortality rates for patients with cirrhosis among different hospitals and medical centers, suggesting that there are substantial variations in practices and quality of care. These studies have also shown low adherence to national practice guidelines and quality measures.^3^,^4^,^5^,^6^,^7^,^8^ Multiple strategies, such as integrated care models, population health management, mandatory gastroenterology/hepatology consultation, education outreach, discharge bundles, and standardized templates,^9^,^10^ have been trialed to improve the quality of inpatient cirrhosis care. These approaches, however, may be cost-prohibitive and not scalable or generalizable across different institutions.

Clinical decision support (CDS) systems are informatics-driven interventions in the electronic health record (EHR) that provide clinicians with patient-specific information or recommendations.^11^,^12^,^13^,^14^,^15^ They are thought to change provider behaviors through improvements in workflow processes, presentation of pertinent information, and facilitation of clinical decision-making. They are an attractive strategy to improve guideline adherence due to potential low costs and scalable deployments across institutions. Several CDS systems have been demonstrated in the management of chronic liver diseases,^16^ including best practice advisory alerts and clinical dashboards for the treatment of chronic hepatitis B and C,^17^,^18^ workflow support for metabolic dysfunction–associated steatotic liver disease (formerly nonalcoholic fatty liver disease),^19^ and medication management.^20^,^21^ Adoptions and implementations of CDS for cirrhosis management, especially inpatient management, have been limited. The reasons are multifactorial: (1) cirrhosis and its complications impact multiple nonhepatic organ systems, (2) the delivery of guidelines-based cirrhosis care requires integrating disparate sources of EHR data, and (3) clinical workflows for cirrhosis care are complex and require coordination of multiple clinical specialties.^11^,^22^,^23^

To overcome these issues, human-centered design (HCD) is an approach that systematically engages with and prioritizes the needs and preferences of end-users in the development of a service or intervention. By addressing and emphasizing functional and usability considerations, HCD helps to construct interventions that end-users will *actually* use.^24^,^25^ In this study, we applied principles from these approaches to design the interface and features for a CDS system for cirrhosis management, called *CirrhosisRx*.

## METHODS

**FIGURE 1:**
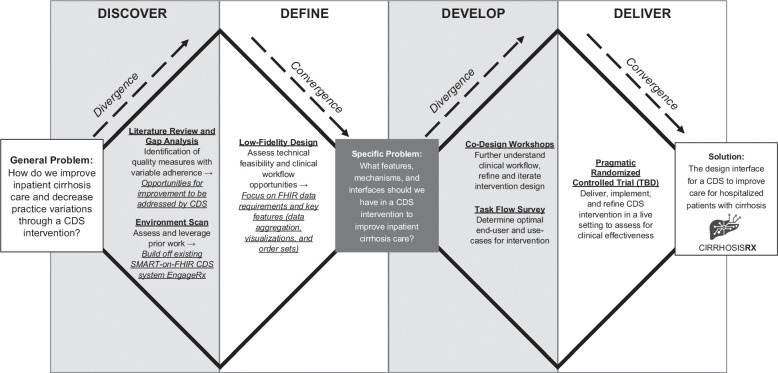
Summary of HCD for the features and interface of *CirrhosisRx*. Abbreviations: CDS, clinical decision support; HCD, human-centered design; SMART-on-FHIR, Substitutable Medical Applications and Reusable Technologies on Fast Health Interoperability Resources; TBD, to be determined.

The activities described in this study were authorized by the Institutional Review Board at the University of California, San Francisco under Study #21-35233. Before embarking on this study, our team had conducted an evaluation of prior literature related to addressing quality gaps in cirrhosis care and determined a priori to create a CDS system for inpatient cirrhosis management. In this stage of the HCD process, therefore, we focused on designing the features and interface of the CDS system that could potentially address the needs of end-users. We organized our activities according to the British Design Council’s “Double Diamond Model.” We chose this model to frame and guide our HCD activities due to its broad acceptability in the design community and its wide applicability to various design problems, including software development.^26^,^27^ The Double Diamond Model divides the design process into 4 main phases (Discover, Define, Develop, and Deliver).^28^ Our activities covered the first 3 phases of the “Double Diamond Model” and are summarized in Figure 1.

### Discovery: Literature review and gap analyses

We first evaluate gaps in the inpatient care of patients with cirrhosis through a review and evaluation of quality measures published by national practice societies, notably from the American Gastroenterological Association (AGA) and the American Association for the Study of Liver Diseases (AASLD), to determine potential clinical actions to be enacted by *CirrhosisRx*. We identified 5 quality measures published by the AGA and AASLD that were applicable to inpatient cirrhosis care and could be subject to intervention by CDS systems—either through presentation, aggregation, or organization of relevant clinical information and/or recommended order sets for standardizing management.^29^,^30^ We then conducted a targeted literature review to determine the existing compliance with these 5 quality measures and found significant variations and opportunities for improvement (Table 1).^3^,^4^,^5^,^6^,^7^,^8^

**TABLE 1:** AGA/AASLD-recommended guidelines and quality measures applicable for inpatient cirrhosis care and previous adherence rates in the literature

#	Description	Numerator (admissions with action performed)	Denominator (addressable admissions)	Rates in previous literature
1	Patients with ascites should receive a diagnostic paracentesis if admitted for ascites or HE	#Admissions with paracenteses	#Admissions with ascites or HE	22%–58%^4^,^7^,^9^
2	Patients who are admitted with or develop GI bleeding should receive antibiotics within 24 h	#Admissions with antibiotics within 24 h	#Admissions with GI bleeding	39%–49%^4^,^7^
3	Patients with SBP should receive empiric antibiotics and i.v. albumin within 12 h	#Admissions with antibiotics/albumin within 12 h	#Admissions with SBP	72%–77%^4^,^7^
4	Patients who present with upper GI bleeding should receive upper endoscopy (EGD) within 12 h	#Admissions with EGD within 12 h	#Admissions with GI bleeding	85%^8^
5	Patients with HE should receive lactulose	#Admissions with lactulose	#Admissions with HE	58%–95%^4^,^5^,^6^

Abbreviations: AASLD, American Association for the Study of Liver Diseases; AGA, American Gastroenterological Association; EGD, upper endoscopy; GI, gastrointestinal; SBP, spontaneous bacterial peritonitis.

### Discovery: Environment scan

To expedite the technical aspects of the design of *CirrhosisRx*, we leveraged prior work, specifically the design and technical expertise accumulated from *EngageRx*, a hypertension management CDS system designed and implemented at our institution. *EngageRx* was developed to help clinical providers with hypertension management in the outpatient setting through the automated incorporation of biometrics and medication records recorded in our EPIC-based EHR system.^31^ *EngageRx* was built on the Substitutable Medical Applications and Reusable Technologies on Fast Health Interoperability Resources (SMART-on-FHIR) application programming interface, which is a set of open specifications for creating applications that could be implemented in any compliant EHR system.^32^ SMART-on-FHIR–enabled portability of CDS systems is particularly important for dissemination to other health and EHR systems.

### Define: Wireframe

**FIGURE 2:**
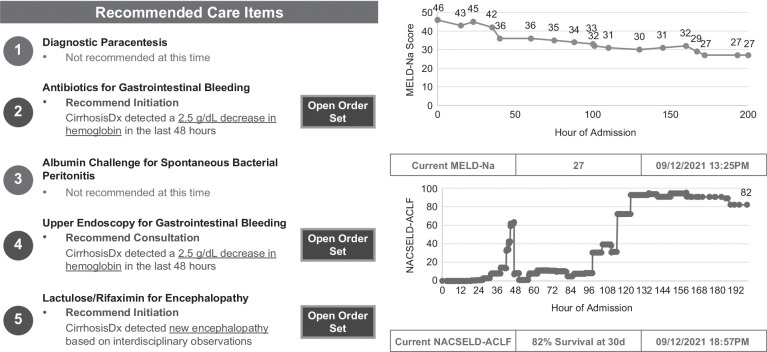
Initial wireframe for *CirrhosisRx* at the time of technical feasibility assessment. Abbreviations: MELD-Na, Model for End-Stage Liver Disease Sodium Score; NACSELD-ACLF, North American Consortium for the Study of End Stage Liver Disease Acute on Chronic Liver Failure Score.

In this phase of the work, we assessed the technical feasibility and identified clinical workflow opportunities. We convened 2 initial focus groups via video teleconferencing that included 2 clinicians, 2 user-experience designers, 1 informaticist, and 2 programmers to brainstorm potential features and design a “ wireframe” of the proposed *CirrhosisRx* CDS system. Wireframes are concept sketches or visual blueprints of the interface and include only the most basic content and visuals. The clinical participants described scenarios in which *CirrhosisRx* is intended to be used while technical experts (user-experience designers, informaticists, and programmers) confirmed technical feasibility with regard to data needs. Due to its planned use of SMART-on-FHIR, all data requirements for *CirrhosisRx* were required to conform to the Fast Healthcare Interoperability Resources (FHIR) health information exchange standards.^32^,^33^ Following these workshops, we created an initial *CirrhosisRx* wireframe that would serve as the basis for our subsequent clinical collaborative-design workshops (Figure 2).

### Develop: Clinical collaborative-design workshop round 1

Following the development of our low-fidelity wireframes, we convened the first of 2 rounds of co-design workshops via video conference. A collaborative-design (or “co-design”) workshop is defined as one where participants (and potential end-users) work with the design team to discuss existing practices, jointly explore ideas, and modify mock-ups or prototypes.^24^,^34^,^35^ We recruited 20 clinicians of various training backgrounds including internal medicine residents; gastroenterology and advanced/transplant hepatology fellows; attending hospitalists, gastroenterologists, and hepatologists; and advanced practice practitioners. These clinicians practiced at a community-affiliated academic practice, an academic medical center, and a Veterans Health Administration hospital. Recruitment was conducted through a snowball sampling method in which existing participants would refer other potential participants to the investigators. Approximately half of the clinicians were self-identified generalists, and the other half were specialists. We convened four 60-minute co-design workshops through video teleconferencing with screen sharing enabled in the first round with 5 to 7 participants (with a rough half/half mix in generalists and specialists) in each. We conducted the first round of co-design workshops with semi-structured interviews. To elicit information and reach a consensus, we utilized the nominal group technique, in which participants wrote down initial thoughts/responses to questions, and shared them with the broader group, followed by discussion and consensus building, to reach final conclusions.^36^

Specifically, we asked the clinical participants regarding the following topics:

1. Approaches and workflows for managing a patient who presents to the hospital with newly diagnosed cirrhosis or a complication of cirrhosis.
2. Sources and location of data within the EHR to help synthesize clinical information and methods of retrieval.
3. Identify difficulties in accessing relevant information within the EHR system that would help them with clinical decision-making.
4. Features of a potential CDS system that would ease difficulties in accessing information in each user’s respective EHR system.

At the near end of each of the 4 co-design workshops, we shared the most updated version of the design prototype for group feedback. We used the iteratively updated design prototype to discuss specific appearances of CDS elements and proposed features, such as data tables, visualizations, and recommended order sets. After each co-design workshop, we reviewed the teleconference recordings along with notes transcribed during the session. We then conducted thematic analyses of the recordings and notes from each workshop to determine the most pertinent recommendations and features to be included in the next iteration of the design prototype.

### Develop: Individual task-flow surveys

Following the 4 co-design workshops in round 1, we sent all participants individual task-flow surveys (Table 2). Task flow in user-experience generally refers to the sequence of steps or actions that a user must go through to complete a specific task. The purpose of a task-flow survey is to elicit the users’ experiences and feedback as they move through certain aspects of the design. In the case of *CirrhosisRx* design, our survey concerned how users would move through and use the individual features, such as the data tables, visualizations, and recommended order sets, of the CDS. Our goal was to elicit input regarding how each feature would be integrated into their workflow for cirrhosis care and a rating of whether they would be using each individual feature.

**TABLE 2:** Task-flow survey questions and responses (N=17 respondents)

Task-flow survey question	Description	Summary statistic or representative responses/quotes
For the “Summary of Complications” feature, how likely are you to incorporate this feature in your daily workflow?	Rated on a Likert Scale 1–5 corresponding to “Extremely Unlikely” to “Extremely Likely”	4.71 (SD 0.46)
For the “Summary of Complications” feature, what information did you find useful in helping you with your workflow?	The “Summary of Complications” refers to the organization of clinical date into 6 decompensation domains (eg, encephalopathy, varices, ascites, renal, nutrition, and infection)	Representative Generalist Comments: “Having most of what you need for a cirrhosis patient in one place.” “I really appreciated that it had the current medications and most recent imaging. Saves me a lot of time. Also, the flow helps me make sure I remember to address all these issues.” Representative Specialist Comments: “Helpful features included the recent lab values and the ‘last/most recent’ pertinent study pertaining to each individual section. Additionally, the current medications are helpful as well.” “I love that it prompts doses of medications and suggests orders. This is going to help the interns and streamline the order process. It will make our rounding faster, and patients can get started on medications ASAP.”
For the “Summary of Complications” feature, what other information would be useful to you to make what kinds of decisions?		Representative Generalist Comments: “Would be helpful to have decision support guidance on, say, discontinuing non-selective beta blocker if they have SBP with a link. Just some nudges.” “I like this how it is. I would say that the outgoing links seems are consequences of complications, that is, esophageal varices, has an outgoing link to EV bleed order set.” Representative Specialist Comments: “If space permits, can consider time/date stamp on when the current medication was ordered/changed (e.g., helpful with the antibiotics section as it would allow for day #, as some of patients may have several antibiotics on board), thus, would help provide a better bird’s eye glance.” “I would move infection up in the order because that is applicable to every cirrhosis patient. Less so the varices (which I think should be moved down). Personally, in terms of how common, I would order it as: Infection, AKI, Ascites, HE, Varices.” “It would be nice to add MAP to the “Renal Dysfunction” section as a way to help assess the likelihood of HRS and to assess treatment response once it is initiated.”
For the “Laboratory Trend” feature (graphs), how likely are you to incorporate this in your workflow?	Rated on a Likert Scale 1–5 corresponding to “Extremely Unlikely” to “Extremely Likely”	4.30 (SD 0.75)
For the “Laboratory Trend” feature (graphs), what information did you find useful in helping you with your workflow?	The “Laboratory Trend” refers to visualizations and graphs of calculated risk scores (MELD)	Representative Generalist Comments: “As a hospitalist, I don’t use MELD-Na on a daily basis.” “The trend is helpful to have the provider begin considering the urgency of transplant evaluation and, perhaps, guide conversations re: prognosis with patients and families.” “It is nice that meld is calculated for the user and displayed in a linear time series. I would find this helpful for a quick look at how the patient is doing from MELD standpoint.” Representative Specialist Comments: “I like the various trend selections (quarter, month, week).” “I’m not sure about this. I feel like big trends are useful—but worried that I will interpret small changes are more clinically significant than they really are.”
For the “Laboratory Trend” feature (graphs), what other information would be useful to you to make what kinds of decisions?		Representative Generalist Comments: “The trend information is very helpful, and when looking at the complications, may be helpful in titrating medications for primary or secondary prophylaxis of complications. So, as an example, if able to have a similar type of graph for the trend of the creatinine (to assess for likelihood of HRS), potassium (to titrate dosing of furosemide/spironolactone), and sodium (to see which direction it has been trending), that may benefit decision making.” Representative Specialist Comments: “Additional ways to improve this section is for additional drop-down selection of different labs (e.g., Hgb, Cr, AST, ALT, Tb, etc.) along with the MELDNa composite score (though can also complete in the results/lab section as flow chart).” “An AFP trend would be helpful if the patient had HCC.”
For the “Laboratory Results” feature, how likely are you to incorporate this in your workflow?	Rated on a Likert Scale 1–5 corresponding to “Extremely Unlikely” to “Extremely Likely”	4.35 (SD 0.68)
For the “Laboratory Results” feature, what information did you find useful in helping you with your workflow?	The “Laboratory Results” refers to the data table feature of multiple commonly used laboratory studies used in the management of cirrhosis	Representative Generalist Comments: “It’s helpful to see all the most important relevant data.” “Need a few values if we have them. If the patient is new of course then still at least two columns are kind of necessary to understand the patient’s outpatient baseline. On that note, it is actually helpful to have color coding in the labs based on the setting of the encounter they are attached to. If all outpatient labs are green and inpatient labs are blue, helps me see quickly that they patient suddenly has new hyponatremia.” Representative Specialist Comments: “Not sure yet. I’m so used to the interface of labs where I normally look at them that I’m not sure this is that useful.” “The 30-day historical range is good, though for creatinine/bili/sodium/INR it would be nice to also have a sense of the trend in terms of which direction each lab value has been going in.”
For the “Laboratory Results” feature, what other information would be useful to you to make what kinds of decisions?		Representative Generalist Comments: “When seeing such a table it can be difficult to have to go through line by line. Would be helpful if it’s broken down based on CBC/BMP/Fluid etc. That way you can find what you need quickly and note what is missing.” “Some other lab complications are useful for us to know about especially if they can be explained by the cirrhosis: cytopenias especially come to mind.” Representative Specialist Comments: “The platelet count would be useful!”
From the list below, which best describes your thinking about the overall concept?		“This would be slightly better than what I am currently using”—53% “I need it because nothing else solves this problem”—47%
Who else do you think this tool would be useful for?		Generalists (Hospitalists, Primary Care Providers, Trainees, and etc.)—75% Specialists (Gastroenterologists, Hepatologists, and etc.)—25%

Abbreviations: MELD, Model for End-Stage Liver Disease; MELD-Na, Model for End-Stage Liver Disease Sodium Score.

### Develop: Clinical co-design workshop round 2

Utilizing the individual feedback that we received from the task-flow surveys, we refined the design prototype and conducted a second round of co-design workshops with all participants from round 1. We convened two 60-minute co-design workshops through video teleconferencing with screen sharing enabled in the second round with 7 to 10 participants (with a rough half/half mix in generalists and specialists) in each. During the round 2 co-design workshops, the design team reviewed the prototype thus far and reported high-level summaries of the task-flow survey results. We then used the consensus-building method of collaborative mapping to obtain additional feedback on the design by asking users to describe, map, and chart out their potential interactions with the design prototype through the screen-sharing functionalities of the teleconference platform.^37^ Specifically, we asked participants to give detailed feedback on the positioning of various features and elements within the design. It was through this process that we also discussed where the final design would be located within the EHR system and how it might best be accessed by clinicians during their usual workflow. As in the first round of co-design workshops, we reviewed the teleconference recordings along with notes transcribed during the workshop for thematic analyses at the conclusion of each workshop.

## RESULTS

**FIGURE 3:**
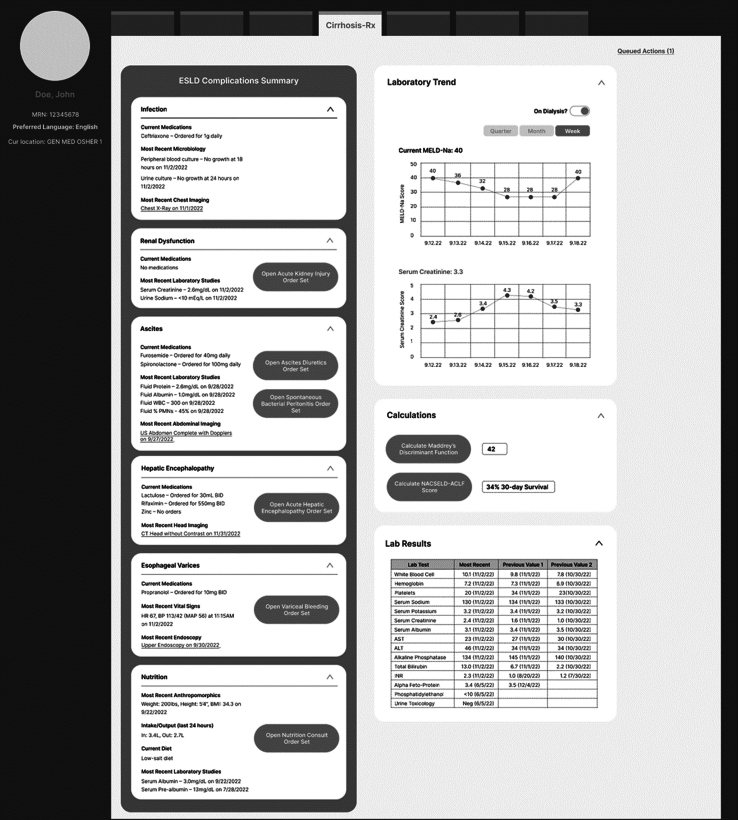
Final *CirrhosisRx* design prototype after co-design workshops. Abbreviations: MELD-Na, Model for End-Stage Liver Disease Sodium Score; NACSELD-ACLF, North American Consortium for the Study of End Stage Liver Disease Acute on Chronic Liver Failure Score.

The final interface and feature design prototype for *CirrhosisRx* from our co-design workshops is presented in Figure 3. This HCD-driven co-design exercise revealed several key insights that affected *CirrhosisRx’s* ultimate design. During the technical feasibility, our use of the FHIR standard (to be compatible with SMART-on-FHIR) set limitations on potential features to be included in the CDS system. For instance, structured data, such as discrete labs, vital signs, and pro-forma documentation with established Logical Observation Identifiers, Names, and Codes (LOINC) codes, were the most feasible to incorporate. Unstructured data, such as clinical notes and pathology reports, were significantly harder to parse and therefore were not considered for incorporation, at least in the initial version of *CirrhosisRx*. These initial technical feasibility reviews set boundaries to guide further discussions with clinicians and end-users in the 2 rounds of clinical co-design workshops.

The 2 rounds of co-design workshops gave insights collected through consensus building (nominal group technique and collaborative mapping) and thematic analyses on which features were most desirable for end-users (Table 3). For instance, our team had initially envisioned automated detection of clinical events, such as gastrointestinal bleeding, based on real-time EHR data as a potential feature for *CirrhosisRx*. This vision, however, was abandoned due to the potential for inaccuracies in algorithms used in the CDS. Moreover, participants reported that “less complex” information aggregation tasks, such as centralizing relevant clinical information into one location, were the most important features of the CDS. With regard to the organization of clinical information—our users strongly preferred organization into 6 commonly recognized “decompensation domains” or generally encountered clinical scenarios in the management of hospitalized patients with cirrhosis, for example, encephalopathy, varices, ascites, renal, nutrition, and infection. Four of these domains, namely encephalopathy,^38^ esophageal varices,^39^ ascites, and renal dysfunction,^40^ are widely accepted as “traditional” decompensations of cirrhosis. Among the user groups, however, there was increasing recognition that nutrition and infection are also important decompensation domains. It was thought that nutrition and infection may become the focus of future quality measures.^41^,^42^,^43^,^44^,^45^

Visualizations, such as longitudinal displays of commonly used risk stratification scores like the Model for End-Stage Liver Disease score,^46^ were found to be helpful but not absolutely necessary: many subspecialized gastroenterologists and hepatologists do not actively calculate Model for End-Stage Liver Disease during their information-gathering. On-demand calculators for other well-known risk scores, but less commonly used than Model for End-Stage Liver Disease, such as Maddrey’s Discriminant Function and the Lille Score,^47^ were appreciated by end-users.

During our co-design workshops and task-flow survey, a major theme that emerged was a significant dichotomy among provider groups (generalists vs. specialists) with regard to desired features (Table 3). For instance, there was mixed feedback regarding the incorporation of info-buttons or links to reference materials for educational purposes. Generalists were largely in favor of such links to established evidence while specialists were less enthusiastic. Eliciting the differences in use-cases and use-workflows also highlighted the importance of identifying the most applicable audience for *CirrhosisRx*. We concluded that *CirrhosisRx* should be focused on the generalist user population rather than specialists who may be far more familiar with recommended guidelines for the inpatient management of cirrhosis.

**TABLE 3:** Key findings and themes extracted from co-design workshops for *CirrhosisRx*

Key theme	Description
CDS logic accuracy	Accuracy of data presented to the user is paramount—inaccurate data or guidance would lead to abandonment of the application.
Data organization	Inpatient cirrhosis care is often framed in terms of each decompensation; therefore, information should be organized by “decompensation domains.”
Notifications	Best practice advisories and “pop-ups” are not desirable, if decision support is recommended, the notification should be a “nudge”
Customizability	Customizability based on provider type or individual user to have different features would be ideal if technically feasible.
Automated calculations	Automated calculation of commonly utilized risk stratification scores, such as Maddrey’s Discriminant Function, Lille Score, and VOCAL-Penn would be helpful
Visualizations	Longitudinal visualization of MELD-Na would be a helpful feature, but calculated risk mortalities within 90 d at each score range would have been more helpful for workflow.
Reference materials	Info-buttons or links to reference materials were favored by generalists, but not specialists.

Abbreviations: MELD-NA, Model for End-Stage Liver Disease Sodium Score.

## DISCUSSION

In this study, we described the HCD process for *CirrhosisRx*, a CDS system intended to improve the inpatient management of cirrhosis and its complications. The process allowed us to identify features that would be relevant for different types of clinical users and workflows. Moreover, our 2 rounds of co-design workshops allowed us to focus on the best-intended user population, which we identified to be generalist practitioners rather than specialists. Finally, discussions in our co-design workshops indicated significant enthusiasm regarding the existing features of the CDS (data tables, visualizations, and recommended order set), but also future features that would be built into *CirrhosisRx.*

To our knowledge, our application of HCD principles to design the features and interface of a CDS system is one of the first for cirrhosis management. There are, however, several limitations to our study. As noted in the methods section, we had decided a priori to focus on the development of a CDS system as the potential solution to inconsistent care and practice variation in the inpatient management of cirrhosis. As such, we only applied the HCD principles and processes to the design of the CDS system and we did not utilize HCD methods in the exploration of alternative strategies for improving inpatient cirrhosis care. Second, the terminology and wording posed in our task-flow survey may have biased responses to positive results. We are aware of this limitation and will carefully construct future surveys and questionnaires related to the evaluation of *CirrhosisRx* to minimize bias and be more neutral. Finally, at the time of the finalization of the prototype design, we did not have a version of *CirrhosisRx* implemented in the testing (protected sandbox) environment in our institution’s EHR to demonstrate real-time integration of the application. We anticipate further iterative testing as we complete software development and production, and move toward live implementation of *CirrhosisRx.*

Despite these limitations, we believe that the future evaluations (both effectiveness and implementation) of *CirrhosisRx* will be pivotal in establishing whether CDS systems for cirrhosis management could: (1) increase recommended care and improve guideline adherence in cirrhosis; and (2) improve hospitalization outcomes, such as 30-day readmissions and in-hospital mortality. Pursuant to this, we are in the process of kicking off a pragmatic randomized controlled trial within our institution to test *CirrhosisRx* in a rigorous manner.

The advent of generative artificial intelligence (GAI) technologies in the past year also opens the potential for their applications in CDS systems. Our group had previously demonstrated several potential use-cases for GAI and large language models in clinical hepatology.^48^ Large language models have been used to refine CDS logic by improving the specificity of alerts in advisory notices.^49^ Initial applications of GAI will likely be in drafting and editing notes and reports, but will ultimately shift toward improving the quality of information for tasks. One potential implementation of GAI technologies in CDS systems, such as *CirrhosisRx*, could be the real-time clinical decision augmentation (eg, recommendations for labs, imaging, or medication orders for next steps in workup or management) detected within the text of the written assessment and plan. A feature like this would be similar to the “auto-complete” function seen in word processing software or search engines, except this would be implemented in a clinically focused context. The future integration of GAI technologies within CDS for clinical application, however, will need to consider issues concerning patient privacy and ongoing concerns about confabulation and hallucinations observed in large language models.^48^,^50^ Moreover, there are also outstanding questions regarding liability and whether GAI clinical tools would be regulated medical devices. At this time, *CirrhosisRx* is not designed or envisioned to include GAI capabilities or algorithms.

In conclusion, this is one of the first studies to describe the process for incorporating HCD-driven design principles into prototype creation of a CDS for the management of cirrhosis. The co-design processes with potential users significantly altered planned features for *CirrhosisRx*, the overall design of the interface, and likely improved the overall usability of the *CirrhosisRx* application. We hope that this work provides a framework for the creation and design of future EHR-based interventions, such as those in the future with GAI capabilities, in hepatology care.
